# Does Oral Ingestion of *Piper sarmentosum* Cause Toxicity in Experimental Animals?

**DOI:** 10.1155/2013/705950

**Published:** 2013-10-08

**Authors:** Maizura Mohd Zainudin, Zaiton Zakaria, Nor Anita Megat Mohd Nordin, Faizah Othman

**Affiliations:** ^1^Department of Physiology, Faculty of Medicine, Universiti Kebangsaan Malaysia Medical Center, Jalan Raja Muda Abdul Aziz, 50300 Kuala Lumpur, Malaysia; ^2^Department of Basic Medical Sciences, Kulliyyah of Medicine, International Islamic University Malaysia, Jalan Sultan Ahmad Shah, Bandar Indera Mahkota, 25200 Kuantan, Pahang, Malaysia; ^3^Department of Anatomy, Faculty of Medicine, Universiti Kebangsaan Malaysia Medical Center, Jalan Raja Muda Abdul Aziz, 50300 Kuala Lumpur, Malaysia

## Abstract

The prevalence of diabetes mellitus has reached epidemic proportion in Malaysia and worldwide. Scientific studies have shown that herbal plant *Piper sarmentosum* exhibits an antidiabetic property. Despite the extensive usage and studies of this herb as alternative medicine, there is paucity of the literature on the safety information of this plant. Thus, the present study aimed to observe the subacute toxic effects of *Piper sarmentosum* aqueous extract (PSAE) on the haematological profile, liver, and kidney in rats. The extract was administered by oral gavage to 6 male and female *Sprague Dawley* rats in daily dose of 50 mg/kg, 300 mg/kg, and 2000 mg/kg for 28 consecutive days. The control group received normal saline. General behavior of the rats, adverse effects, and mortality were observed for 28 days. The haematological and biochemical parameters were determined at baseline and after the treatment. PSAE did not show abnormality on the body weight and gross observation of internal organs. The haematological, biochemical and histopathological profiles showed minimal changes and variation within normal clinical range except for significant increase in serum potassium level that suggests the need of regular monitoring. Nevertheless, these findings suggested that PSAE up to 2000 mg/kg/day did not show subacute toxicity in *Sprague Dawley* rats.

## 1. Introduction

Diabetes mellitus (DM) is chronic disease that leads to severe sequelae with multiple organ involvement. The prevalence of DM is currently on the rise in Malaysia and worldwide. WHO has estimated that 366 million people will have DM by the year 2030. The Malaysian National health and Morbidity Survey (NHMS) has shown that the prevalence of DM in individual age of 30 years and above in Malaysia has increased from 6.3% in 1986 to 14.9% in 2006. Various efforts have been done focusing on the advancement of the therapeutic approach to prevent the occurrence of DM, optimization of blood sugar level, minimization of the symptoms and complications, and prolongation of survival rates in patient with DM.

In phytomedicine, various studies have recognized that *Piper sarmentosum* Roxburgh (PS) has high antioxidant activity and also exhibits the antidiabetic property [1, 2]. PS is known as *kaduk* in Malay, a herb that belongs to the Piperacea family. It is widely distributed in tropical countries in southeast Asia, northeast India, and China [3]. It is a creepy terrestrial herb, with an average height of 20 cm, and grows in shaded areas. The leaves are heart-shaped and green in colour. The flower is white in colour and bar-shaped. The flowers will develop into fruits like a berry. PS has been widely used as both cuisine and traditional remedy [2] in the treatment of diabetes mellitus [4], cough, toothache, fungal infection on the skin, asthma, and inflammation of the pleura [5, 6].

Experimental study in the late 1980s had shown that the aqueous extract of PS leaves helps to reduce blood glucose level in the Alloxan-induced diabetic rabbits. Nevertheless, the extract did not affect the blood sugar level of the normal fasted rabbit [7, 8]. Other studies showed that the water extract of the whole plant of PS exhibits antioxidant effect and hypoglycaemic effect on Streptozotocin-induced diabetic rats [2, 4]. Previous research had shown that PS has cardiovascular protective effects. It showed the ability to increase nitric oxide production in human umbilical vein endothelial cells (HUVECs) [9, 10] and antiatherosclerotic property. In 2012, a study showed that PS aqueous extract was able to remodel ultrastructure stability of the cardiovascular tissue in the Streptozotocin-induced diabetic rats [11]. The other laboratory study showed that PS has a potential as having antiamoebic [12], larvicidal [13], anti-inflammatory, antipyretics [14], antituberculosis [15], and anticarcinogenic effect [16]. It also has the ability to reduce visceral fat, maintain blood glucose level, and reduce 11*β*-hydroxysteroid dehydrogenase in obese rats [17, 18].

Despite extensive usage and study of this herbaceous plant, no comprehensive study on its toxic effects has been reported. Previous study had shown that the LD_50_ of PS whole plant aqueous extract was more than 10 g/kg per oral in rats [4], while PS leaves' methanol extract LD_50_ was more than 5 g/kg in mice [14]. The present study was carried out to determine the general toxic effects and the dose-related effects specifically focusing on the kidney and liver following subacute oral dosing of PS aqueous extract in rats to provide information on its safety and guidance for selecting a safe dose of PS for its use in medical practice.

## 2. Materials and Methods

### 2.1. Plant Material

The leaves of PS were collected from a palm oil farm in Pahang, Malaysia, and authenticated by Mr. Kamaruddin, a plant taxonomist from the Herbarium Unit, Forest Research Institute Malaysia (FRIM). A voucher specimen (PID 240812-17) was deposited in the Department of Physiology, Universiti Kebangsaan Medical Center, Kuala Lumpur, Malaysia.

### 2.2. Preparation of Aqueous Extract of *Piper sarmentosum* Leaves

The fresh leaves of PS were washed with tap water and oven-dried at temperature of 50°C for 36 hours. The dried leaves were cut into small pieces. Then, 10 grams of the dried PS leaves was added to 900 mL of distilled water, and it was boiled at 80°C for 3 hours for the extraction process. The water extract was then concentrated, followed by freeze-drying into powder form. The powdered extract was stored at 4°C until usage. In this study, the extract was prepared according to the previous studies protocol [15, 16].

### 2.3. Experimental Animals

Both male and female *Sprague Dawley* rats weighing 200 g ± 20% body weight, obtained from Animal Unit of Universiti Kebangsaan Malaysia were used in this study. All rats were quarantined for seven days before treatment to allow acclimatization. They were housed in polypropylene cage (one per cage) and kept in temperature of 22°C ± 3°C and humidity constant at 50 to 60% with controlled lighting that provides 12-hour light-dark cycle. All procedures in the experiment were carried out in accordance with the institutional guidelines for animal research of UKM with animal ethics approval number: PP/FISIO/2010/ZAITON/17-MARCH/299-APRIL-2010-DECEMBER-2011. Water and rat chow (Gold Coin, Malaysia) were given ad libitum, and all animal manipulations were carried out in the morning to minimize the effects of circadian rhythm. Treatments of *Piper sarmentosum* aqueous extract (PSAE) were given at constant concentration at different volumes according to the dosage and body weight, respectively. The volume was not more than 2 mL/100 g body weight per dose.

### 2.4. Toxicological Evaluation of the *Piper sarmentosum* Aqueous Extract

Forty-eight healthy *Sprague Dawley* rats of both sexes were divided into three treatment groups and a control group consisting of six male and female rats. Pretreatment blood taking was done via retroorbital sinus bleeding method. Sera were collected and sent for haematological and biochemical analysis. Treatment groups were given the extract with different doses of 50, 300, and 2000 mg/kg body weight, while the control groups were given normal saline according to OECD guideline [19]. The body weights were measured and recorded at baseline and then weekly. The water and food intake were determined daily. The extract was administered using a straight, ball-tipped stainless steel feeding needle for 28 consecutive days. At day 28, the rats were anaesthetized with a cocktail of ketamine, xylazil, and zolatil. Then, rats were sacrificed and necropsy was performed. Post treatment blood samples were obtained via intracardiac puncture method for haematological and biochemical profile examination.

Serum was collected from a blood sample that had been centrifuged at 13000 rpm for ten minutes. Blood samples were sent to the Pathology & Clinical Laboratory (M) Sdn. Bhd. for analysis. The haematological parameters including haemoglobin, red blood cell, pack cell volume, and white blood cell were analyzed by auto analysis Sysmex Kx-21 Haematology Cell Counter (SN:A7667)-ISO no. KL/2006/P/0001. The renal and liver biochemical profiles were analyzed by auto analysis machine Advia 2400 Chemistry Analyser (1) (SN:CA12420030)-ISO no. 2005/P/0006. The renal biochemical profile includes the serum urea, creatinine, uric acid, and the electrolytes (sodium, potassium, and chloride), while the liver biochemical profile consists of the total protein, albumin, globulin, total bilirubin, alkaline phosphatase (ALP), aspartate transaminase (AST), alanine transaminase (ALT), and gamma glutamyl transferase (GGT). The histological sections of the liver and kidney tissues were prepared by haematoxylin and eosin (H&E) staining method at the Department of Anatomy, UKM, Malaysia. 

### 2.5. Statistical Analysis

The results are expressed as mean ± standard error of the mean (SEM). One-way analysis of variance (ANOVA) was used to compare between and within group comparison while Student's *t*-test was used for paired comparison. 95% level of significance (*P* < 0.05) was used for the statistical analysis.

## 3. Results

### 3.1. Changes in Clinical Observation, Body Weight, Internal Organ Weight, and Water and Food Consumption

There were no PSAE treatment-related mortalities recorded in rats after 28 daily dosing of PSAE. None of them showed any obvious morbidity or clinical symptoms of toxicity such as changes in the skin and fur, eyes, and mucus membrane (nasal), respiratory rate, autonomic effects (salivation, perspiration, and piloerection), and central nervous system (ptosis, drowsiness, abnormal gait, tremors, and convulsion) throughout the experimental period.

**Figure 1:**
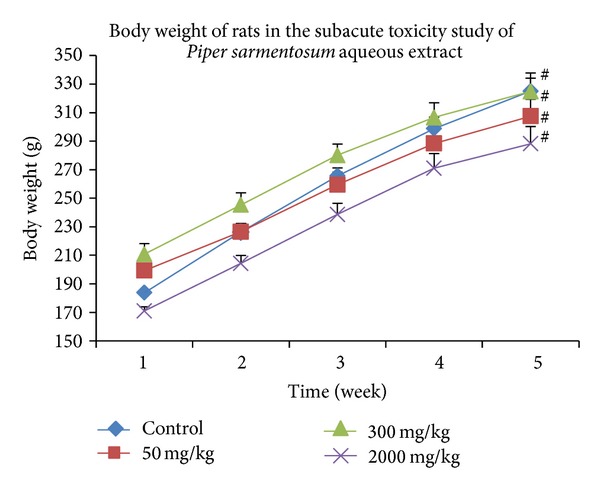
Showing changes in the body weight of rats in various groups throughout the study period. Data were expressed as mean ± SEM. *P value <0.05 is significant compared to control group; ^#^ *P* value <0.05 is significant compared to baseline value; ^*β*^ *P* value <0.05 is significant compared to other groups.

The study showed that rats in both control and treated groups attained significant weight gain (35 to 43%, *P* < 0.05) after 28 days of the experiment compared to baseline weight. However, all groups did not show significant difference in weight in each week throughout the study as compared to control and the other treatment groups (Figure 1). The food and water consumption of male and female rats in both control and treated groups was fluctuating within a constant range throughout the study. The results showed no significant differences in treated groups as compared to control or within group (Table 1).

It was found that the mean relative weights of liver for female group treated with PSAE at the dose of 2000 mg/kg/day were higher than the control and the other treated groups but statistically not significant. For the male rats, there were no significant differences in the mean liver weight in all groups. The mean of the kidney weights of the male rats treated with PSAE of 300 mg/kg was lower than the control and the other treatment groups but showed no significant difference. The mean relative weights of the ovary of rats treated with PSAE were lower compared to the control group but did not show significant difference. The other organs such as spleen, lungs, and testis showed no significant difference that is consistent with the body weight variation (Table 2).

### 3.2. Changes in Haematological Profile

The haemoglobin and pack cell volume (PCV) level in male rats treated with 2000 mg/kg were the lowest at baseline and significant (*P* < 0.05) when compared to control. However, the post treatment levels of both haemoglobin and PCV showed no significant difference when compared to control, baseline, and other groups. The platelets of rats of the female control group were significantly (*P* < 0.05) reduced when compared to baseline. The other parameters such as the red blood cell (RBC), mean corpuscular volume (MCV), mean corpuscular haemoglobin (MCH), and mean corpuscular haemoglobin concentration (MCHC) showed no significant difference when compared to baseline, control, and other groups. The white bloods cells of male rats administered 50 mg/kg body weight showed a significant increase (*P* < 0.05) when compared with the baseline. However, there was no significant difference in WBC of all the other groups as compared to control, baseline, and the other groups (Table 3).

### 3.3. Changes in Renal Biochemical Profile

In the male rats, there was a significant increase (*P* < 0.05) in the levels of urea in groups treated with 50 and 300 mg/kg when compared to the baseline level. The rats in the group treated with 2000 mg/kg showed the lowest urea level as compared to the other groups (*P* < 0.05). The serum creatinine level of group 50 mg/kg was significantly higher at baseline (*P* < 0.05) and showed significant reduction after treatment (*P* < 0.05). Besides that, the serum potassium of the control group treated with 300 and 2000 mg/kg, was significantly increased (*P* < 0.05) when compared to the baseline level. However, the serum potassium level of the treated group showed no significant difference when compared to control group. The other parameters such as the uric acid, sodium and chloride levels showed no significant difference when compared to baseline, control, or treated groups. For the female rat's renal biochemical parameters, the urea level of group treated with 300 mg/kg was the lowest at baseline and showed significant increase when compared to other treatment groups. The creatinine level of the group treated with 50 mg/kg was the highest at baseline compared to control and the other groups and significantly reduced (*P* < 0.05) after treatment when compared to baseline. The serum potassium levels of group treated with 50 and 2000 mg/kg increased significantly (*P* < 0.05) when compared to baseline. The other parameters showed no significant changes when compared to the control, baseline, and other groups (Table 4).

### 3.4. Changes in Liver Biochemical Profile

There was a significant decrease (*P* < 0.05) in the total protein levels in group 50 mg/kg in both male and female rats when compared to baseline. The post treatment globulin level of the male rats group treated with 50 mg/kg was the least and showed significant difference when compared to other groups. The baseline level of ALP of the male rats group treated with 50 mg/kg was the lowest while the level of ALT of the female rats was the highest and showed significant difference (*P* < 0.05) when compared to control group. However, there were no significant changes (*P* < 0.05) in total protein, albumin, globulin, total bilirubin, AST, and GGT of the treated groups compared with control in both male and female rats after subacute administration of PSAE (Table 5).

### 3.5. Histopathological Profile of Liver and Kidney

**Figure 2:**
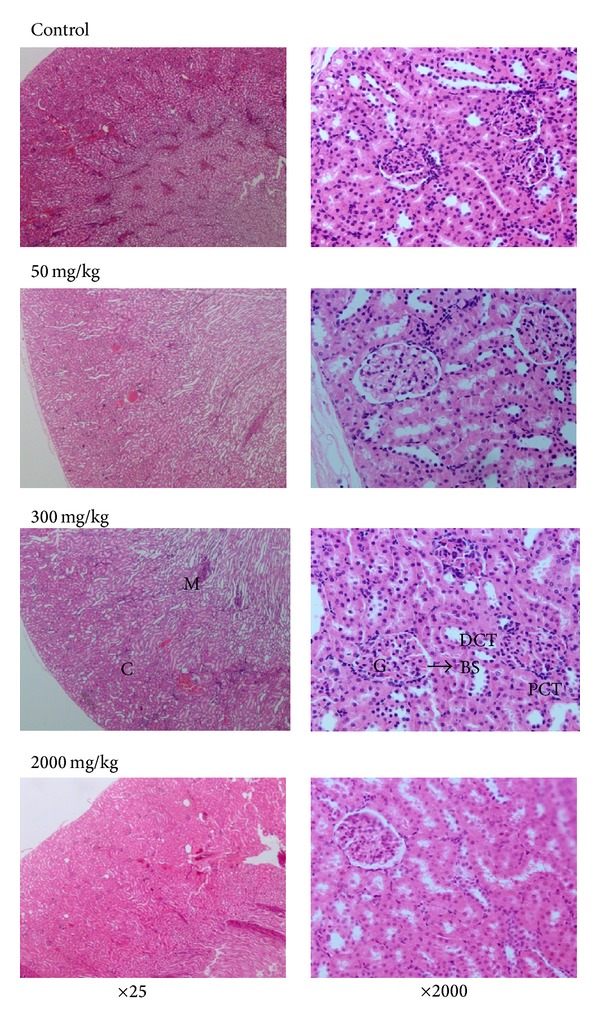
The histology of renal cells with H&E staining method of rats treated with 50 mg/kg, 300 mg/kg, and 2000 mg/kg of *Piper sarmentosum* aqueous extract and normal saline for 28 consecutive days at 25 and 200 times magnification. There were no significant changes in the structure of the kidney cells observed in the histological section of the kidney tissues of the treatment groups. G: glomerulus; BM: basement membrane; PCT: proximal convoluted tubule; DCT: distal convoluted tubule; C: cortex; M: medulla; BS: Bowman's space.

The histology section of the kidney for the control group by H&E staining showed the normal renal cell architecture. The cortex consists of the glomeruli, blood vessels, tubules, and interstitium. The glomeruli were symmetrical with regular and thin capillary walls. The cells of the nuclei were not overlapping, and there were no clusters of cells or hypercellularity. There were no cells infiltrations in the lumen of capillaries. The medullary part of the kidney showed the renal tubules that were arranged in a normal architecture with minimal interstitium. The medullary artery also had thin intima and endothelial lining. The histological section of the group treated with extract 50 mg/kg and 300 mg/kg portrayed the same picture as the control group. On the other hand, the group treated with PSAE 2000 mg/kg showed a slight increase in the interstitium that might suggest cellular infiltration; however, the nuclei and the renal cell architecture were clearly seen (Figure 2). 

**Figure 3:**
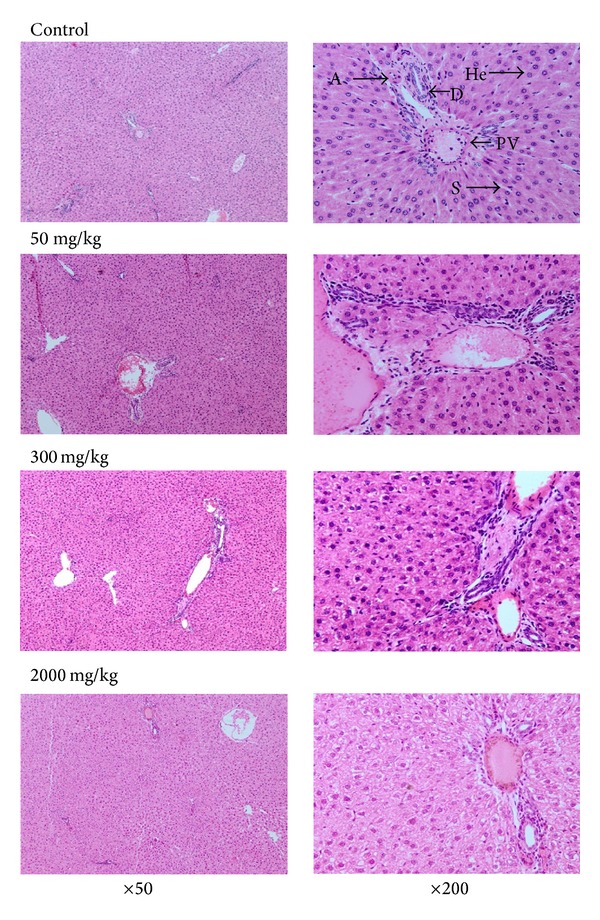
The histology of liver cells with H&E staining method of rats treated with 50 mg/kg, 300 mg/kg, and 2000 mg/kg of *Piper sarmentosum* aqueous extract and normal saline for 28 consecutive days at 50 and 200 times magnification. There were no significant changes showing congestion or destruction of liver cells observed in the histological section of the liver tissues of the treatment groups compared to the control group. PT: portal triad; PV: portal vein; He: hepatocyte; S: sinusoid; A: hepatic artery; D: bile duct.

The histopathological examination of the hepatocytes showed that the control group exhibited normal finding where it showed that the portal triads consist of portal veins, hepatic artery, and bile duct situated at the periphery and the central veins with radiating cords of hepatocytes separated by sinusoids. The hepatocytes were of the same size and polygonal in shape with the nucleus at the center and cytoplasm which was regularly distributed. The treated groups also represent the same picture. There was neither loss of radial arrangement nor thickening or congestion of the sinusoids. There were very minimal cells infiltrations around the portal track. There was no obvious area of necrosis around the central vein (Figure 3).

## 4. Discussion

Herbal plant usage is increasingly becoming more popular as alternative medicine and supplement in the primary health care worldwide [20, 21]. The plan to introduce PS to human should consider the benefits and risks of this herb for the recipient. As PS has been proven to have a tremendous beneficial effect, it is necessary to study its harmful effects before embarking on human studies. In a step to achieve the objective evaluation of the effects of a substance on animals, it is fundamental to look for changes in general behavior, body weight, and haematological profile [22] as such changes are often the first signs of toxicity. Besides, the biochemical profile may also picture the target organ damage. OECD, Guideline 407, has recommended the Repeated Dose 28-day Oral Toxicity Study in Rodents methods to protect animal rights by reducing the number of animals used, reduce suffering, and not to cause death as the endpoint of the study [19].

It is believed that natural plant products are safe, and they has been widely used worldwide for centuries [23]. A previous study showed that PSAE contained high phenolic and flavanoid content in which the main flavonoids are rutin and vitexin [24]. However, it has to be proven safe scientifically before it is used in humans. Another study has showed that the LD_50_ of PS whole plant aqueous extract is more than 10 g/kg per oral in rats [4], while PS leaves' methanol extract LD_50_ was more than 5 g/kg in mice [14]. According to Globally Harmonization System [25], for a substance with LD_50_ of more than 5 g/kg the GHS is unclassified. However, to the best of our knowledge, there is no comprehensive toxicity study which has been performed with PS leaves. The leaves are the most commonly used part of this herb as cuisine and traditional remedy. Hence, it may be beneficial to conduct this study for the human usage of PS later.

The present study provides baseline information for the anticipation of the harmful effect of PS in humans. Besides, the information is also useful to predict the nature of the pharmacokinetics and pharmacodynamics. It may also give a clue to the organ or system that might be affected. The aqueous extract of PS leaves was chosen as it is traditionally taken raw as cuisine and supplement and likely to be patterned from aqueous extract as well. Although the ethanol extract showed more antioxidant compounds, nevertheless the aqueous extract of PS also showed high amount of antioxidant activity.

In the present study, the rats did not show any signs of morbidity and mortality after subacute administration of PSAE. Some plant extracts were reported to cause reduced food intake. However, this extract did not cause reduction in rats' body weight, and food and water intake. In addition, a minimal variation of haemoglobin and WBC count which is still in the normal range suggested that PS did not interfere with the haematopoietic system. Despite showing significant difference statistically, the outcomes of some parameters in the biochemical profile are actually varying within clinical reference range. Important markers for renal impairment such as urea and creatinine did not even double the values similarly with the markers for liver impairment such as ALP, AST, ALT, and GGT [26]. The histopathological examinations also showed very minimal changes that were inconsistent and may be due to technical problems during tissue fixation and processing. The increment of serum potassium level after PSAE administration in this study signifies that a regular monitoring of renal biochemical profile is required upon prolonged intake of PS.

## 5. Conclusion

The findings in the present study suggested that the subacute administration of PS leaves aqueous extract did not cause subacute toxicity in haematological profile, liver, and kidney in *Sprague Dawley* rats. However, further research on the safety of PS involving the other systems such as the reproductive and the central nervous system may be performed in the future. Besides, the subchronic toxicity study of PS may be performed to obtain a complete guidance for the usage of PS in medical practice in human.

## Figures and Tables

**Table 1:** Table showing changes in the food and water consumption of rats throughout the experimental period.

	Control	*Piper sarmentosum* aqueous extract (mg/kg)		
	Week 1	Week 2	Week 3	Week 4
Male				
Water consumption (mL)				
Control	31.0 ± 1.2	36.3 ± 0.8	40.4 ± 1.6	44.6 ± 2.4
PSAE 50 mg/kg	32.4 ± 3.5	38.1 ± 4.4	36.4 ± 4.1	38.8 ± 4.0
PSAE 300 mg/kg	35.0 ± 2.8	35.9 ± 2.8	38.0 ± 3.8	35.3 ± 2.8
PSAE 2000 mg/kg	30.8 ± 5.1	35.0 ± 6.3	34.7 ± 6.3	40.2 ± 7.5
Food consumption (g)				
Control	19.5 ± 0.7	21.9 ± 0.8	23.0 ± 0.8	22.6 ± 1.3
PSAE 50 mg/kg	18.8 ± 1.0	21.3 ± 1.2	21.4 ± 1.0	21.1 ± 0.6
PSAE 300 mg/kg	22.6 ± 1.5	22.7 ± 1.3	22.9 ± 1.0	21.1 ± 1.7
PSAE 2000 mg/kg	16.2 ± 0.7	17.1 ± 1.2	17.1 ± 1.2	19.6 ± 1.3

Female				
Water consumption (mL)				
Control	28.3 ± 3.2	29.7 ± 2.3	32.7 ± 1.8	30.3 ± 1.3
PSAE 50 mg/kg	31.1 ± 3.8	30.5 ± 2.3	33.2 ± 1.8	33.3 ± 0.9
PSAE 300 mg/kg	37.0 ± 3.0	33.8 ± 3.0	38.7 ± 1.9	35.1 ± 0.9
PSAE 2000 mg/kg	29.2 ± 2.8	29.5 ± 2.8	28.9 ± 2.2	29.7 ± 2.0
Food consumption (g)				
Control	14.0 ± 0.7	16.0 ± 0.4	16.1 ± 0.5	15.5 ± 0.6
PSAE 50 mg/kg	12.3 ± 0.4	14.1 ± 0.4	13.6 ± 0.9	13.9 ± 0.3
PSAE 300 mg/kg	15.4 ± 0.7	17.1 ± 0.4	17.1 ± 1.5	18.0 ± 1.0
PSAE 2000 mg/kg	12.6 ± 0.6	15.7 ± 1.8	15.8 ± 0.9	15.7 ± 1.4

Data were expressed as mean ± SEM. *P value < 0.05 is significant compared to control group; ^#^ *P* value < 0.05 is significant compared to baseline value; ^*β*^ *P* value < 0.05 is significant compared to other groups.

**Table 2:** Table showing changes in the relative organ weight of rats throughout the experimental period.

Relative weight (%)	Control	*Piper sarmentosum* aqueous extract (mg/kg)		
	(normal saline)	50 mg/kg	300 mg/kg	2000 mg/kg
Male				
Liver	3.63 ± 0.13	3.60 ± .0.07	3.51 ± 0.12	3.61 ± 0.13
Left kidney	0.35 ± 0.01	0.36 ± 0.02	0.33 ± 0.01	0.34 ± 0.01
Heart	0.39 ± 0.02	0.36 ± 0.02	0.36 ± 0.01	0.32 ± 0.01
Left lung	0.25 ± 0.01	0.25 ± 0.01	0.23 ± 0.01	0.26 ± 0.01
Spleen	0.19 ± 0.01	0.21 ± 0.01	0.18 ± 0.01	0.18 ± 0.00
Left testis	0.49 ± 0.02	0.51 ± 0.03	0.48 ± 0.02	0.53 ± 0.01
Female				
Liver	3.44 ± 0.07	3.42 ± 0.05	3.34 ± 0.14	3.64 ± 0.13
Left kidney	0.34 ± 0.01	0.34 ± 0.02	0.35 ± 0.01	0.34 ± 0.01
Heart	0.37 ± 0.02	0.35 ± 0.01	0.34 ± 0.01	0.37 ± 0.01
Left lung	0.30 ± 0.01	0.28 ± 0.01	0.29 ± 0.02	0.26 ± 0.01
Spleen	0.24 ± 0.02	0.25 ± 0.01	0.25 ± 0.01	0.23 ± 0.01
Left ovary	0.04 ± 0.00	0.03 ± 0.001	0.03 ± 0.001	0.03 ± 0.001

Data were expressed as mean ± SEM. *P value < 0.05 is significant compared to control group;^ #^ *P* value < 0.05 is significant compared to baseline value; ^*β*^ *P* value < 0.05 is significant compared to other groups.

**Table 3:** Table showing changes in the haematological profile of rats throughout the experimental period.

			*Piper sarmentosum* aqueous extract (mg/kg)					
	Control		50		300		2000	
	Pre	Post	Pre	Post	Pre	Post	Pre	Post
Male								
RBC (10^6^/*μ*L)	6.7 ± 0.4	7.2 ± 0.3	6.9 ± 0.1	7.4 ± 0.2	6.8 ± 0.3	7.6 ± 0.2	6.1 ± 0.2	7.0 ± 0.3
HB (g/dL)	13.1 ± 0.3	14.0 ± 0.5	14.2 ± 0.2	14.5 ± 0.4	13.4 ± 0.2	14.4 ± 0.2	12.5 ± 0.2^*β*^	13.5 ± 0.6
PCV	42.8 ± 0.9	45.5 ± 1.9	45.3 ± 0.3	46.4 ± 1.6	44.5 ± 0.9	52.3 ± 5.4	40.0 ± 1.8^*β*^	43.2 ± 2.0^#^
MCV (fL)	67.7 ± 0.7	63.7 ± 0.6	65.7 ± 1.2	63.0 ± 1.0	66.0 ± 1.9	62.0 ± 1.1	66.7 ± 1.2	61.5 ± 0.6
MCH (pg)	20.5 ± 0.3	19.7 ± 0.3	20.7 ± 0.4	19.6 ± 0.4	20.0 ± 0.5	19.0 ± 0.3	20.5 ± 0.4	19.3 ± 0.2
MCHC (g/dL)	30.5 ± 0.2	30.7 ± 0.2	31.7 ± 0.2	31.2 ± 0.2	30.3 ± 0.4	30.5 ± 0.3	31.1 ± 0.6	31.3 ± 0.3
PLT (×10^9^/*μ*L)	1028 ± 53	998 ± 149	956 ± 153	1013 ± 116	1122 ± 82	966 ± 106	760 ± 46	723 ± 162
WBC (×10^3^/*μ*L)	15.7 ± 1.3	11.4 ± 1.8	16.0 ± 1.3	19.1 ± 3.7^#^	18.3 ± 1.8	15.3 ± 2.4	14.0 ± 1.5	10.8 ± 2.3
Female								
RBC (10^6^/*μ*L)	6.8 ± .01	6.5 ± 0.2	6.7 ± 0.3	6.9 ± 0.2	7.2 ± 0.1	7.1 ± 0.2	6.6 ± 0.2	6.9 ± 0.1
HB (g/dL)	14.1 ± 0.2	12.9 ± 0.7	13.3 ± 0.4	13.8 ± 0.3	14.3 ± 0.3	13.8 ± 0.4	12.7 ± 0.4	13.4 ± 0.2
PCV	44.3 ± 0.6	40.4 ± 2.4	42.2 ± 1.8	43.5 ± 0.8	44.7 ± 0.7	42.2 ± 1.2	39.8 ± 1.0	41.7 ± 0.4
MCV (fL)	65.0 ± 0.5	61.4 ± 1.1	63.0 ± 1.0	62.8 ± 0.7	62.0 ± 1.3	59.8 ± 0.6	60.8 ± 1.2*	59.8 ± 0.8
MCH (pg)	20.5 ± 0.2	19.8 ± 0.3	19.8 ± 0.4	20.2 ± 0.3	20.0 ± 0.6	19.6 ± 0.2	19.4 ± 0.5	19.3 ± 0.3
MCHC (g/dL)	31.7 ± 0.2	32.2 ± 0.2	31.5 ± 0.2	31.8 ± 0.2	32.0 ± 0.4	32.6 ± 0.4	32.0 ± 0.5	32.3 ± 0.2
PLT (×10^9^/*μ*L)	998 ± 68	671 ± 65*	773 ± 128	947 ± 55	1086 ± 132	927 ± 60	858 ± 115	842 ± 116
WBC (×10^3^/*μ*L)	19.4 ± 2.3	11.8 ± 2.1	16.4 ± 1.1	10.5 ± 2.5	14.4 ± 2.1	13.6 ± 0.80	15.2 ± 3.0	11.7 ± 2.1

Data were expressed as mean ± SEM. *P value < 0.05 is significant compared to control group;^ #^ *P* value < 0.05 is significant compared to baseline value; ^*β*^ *P* value < 0.05 is significant compared to other groups.

**Table 4:** Table showing changes in the serum renal biochemical profile of rats throughout the experimental period.

			*Piper sarmentosum* aqueous extract (mg/kg)					
	Control		50		300		2000	
	Pre	Post	Pre	Post	Pre	Post	Pre	Post
Male								
Urea (mmol/L)	6.7 ± 0.5	7.6 ± 0.3	5.2 ± 0.1	7.2 ± 0.5^#^	5.8 ± 0.2	7.8 ± 0.5^#^	5.4 ± 0.5	6.2 ± 0.2^*β*^
Creatinine (*µ*mol/L)	24.7 ± 4.9	26.7 ± 1.2	46.7 ± 0.8^∗*β*^	28.2 ± 1.2^#^	30.5 ± 6.0	32.0 ± 1.4	22.3 ± 1.1	33.2 ± 1.6
Uric acid (mmol/L)	0.05 ± 0	0.08 ± 0	0.06 ± 0.01	0.08 ± 0.01	0.07 ± 0.01	0.09 ± 0.02	0.03 ± 0	0.12 ± 0.03
Sodium (mmol/L)	140.8 ± 0.5	139.5 ± 0.7	139.8 ± 0.5	141.3 ± 0.3	140.2 ± 0.8	141.2 ± 0.8	140.7 ± 0.4	140.8 ± 0.4
Potassium (mmol/L)	4.5 ± 0.1	6.3 ± 0.3^#^	5.2 ± 0.2	5.8 ± 0.3	5.2 ± 0.3	6.2 ± 0.2^#^	4.2 ± 0.1^*β*^	6.5 ± 0.2^#^
Chloride (mmol/L)	98.8 ± 0.7	97.5 ± 0.2	101.3 ± 0.6*	100.3 ± 0.4	99.7 ± 0.4	99.3 ± 0.9	99.5 ± 0.3	99.8 ± 0.6
Female								
Urea (mmol/L)	6.6 ± 0.2	8.0 ± 0.4	7.6 ± 0.2	8.2 ± 0.3	6.1 ± 0.3^*β*^	6.8 ± 0.1^*β*^	7.0 ± 0.4	7.7 ± 0.3
Creatinine (*µ*mol/L)	32.5 ± 3.7	28.5 ± 1.7	39.7 ± 1.4^∗*β*^	30.2 ± 1.0^#^	33.8 ± 1.4	31.8 ± 2.3	33.2 ± 2.0	31.3 ± 0.8
Uric acid (mmol/L)	0.10 ± 0.01	0.08 ± 0.01	0.09 ± 0.01	0.08 ± 0.01	0.07 ± 0.01	0.11 ± 0.02	0.04 ± 0.01	0.09 ± 0.01
Sodium (mmol/L)	141.2 ± 1.0	142.5 ± 0.7	140.2 ± 0.9	138.2 ± 0.5	138.0 ± 0.9	141.0 ± 0.9	139.5 ± 1.0	142.5 ± 0.5
Potassium (mmol/L)	4.9 ± 0.3	5.5 ± 0.1	4.8 ± 5.9	5.9 ± 0.2^#^	4.7 ± 0.2	5.3 ± 0.1	4.2 ± 0.2	5.6 ± 0.3^#^
Chloride (mmol/L)	102.2 ± 1.1	99.0 ± 1.3	101.0 ± 0.7	100.7 ± 0.8	99.4 ± 0.4	101.1 ± 1.0	98.5 ± 0.7	101.3 ± 0.9

Data were expressed as mean ± SEM. *P value < 0.05 is significant compared to control group;^ #^ *P* value < 0.05 is significant compared to baseline value; ^*β*^ *P* value < 0.05 is significant compared to other groups.

**Table 5:** Table showing changes in the serum liver biochemical profile of rats throughout the experimental period.

			*Piper sarmentosum* aqueous extract (mg/kg)					
	Control		50		300		2000	
	Pre	Post	Pre	Post	Pre	Post	Pre	Post
Male								
Total protein (g/L)	63 ± 1.1	64.3 ± 1.3	66.7 ± 0.9	63.3 ± 1.5^#^	64.8 ± 1.2	66.2 ± 1.4	65.3 ± 2.3	65.3 ± 1.9
Albumin (g/L)	34.3 ± 0.4	35.3 ± 0.8	34.5 ± 0.4	36.7 ± 0.8	34.2 ± 0.6	35.2 ± 0.7	35.0 ± 0.6	35.2 ± 0.8
Globulin (g/L)	32.0 ± 1.3	29.0 ± 0.9	32.2 ± 1.2	26.5 ± 0.9^*β*^	30.5 ± 0.7	31.0 ± 1.3	30.3 ± 2.1	32.0 ± 0.9
Bilirubin (*µ*mol/L)	1 ± 0	1 ± 0	1 ± 0	1 ± 0	1 ± 0	1 ± 0	1 ± 0	1 ± 0
ALP (iu/L)	531 ± 62	372 ± 44	355 ± 21*	438 ± 39	416 ± 31	332 ± 31	473 ± 17	338 ± 13
AST (iu/L)	131 ± 18	108 ± 7	132 ± 20	147 ± 28	111 ± 8	115 ± 9	105 ± 7	139 ± 19
ALT (iu/L)	60 ± 4	67 ± 3	61 ± 2	84 ± 11	56 ± 4	66 ± 2	63 ± 5	67 ± 4
GGT (iu/L)	0	0	0	0	0	0	0	0
Female								
Total protein (g/L)	74.3 ± 1.3	71.3 ± 1.0	76.2 ± 2.0	66.7 ± 2.1^#^	70.7 ± 1.3	69.7 ± 1.2	71.7 ± 2.3	71.5 ± 2.0
Albumin (g/L)	38.51.0	36.0 ± 0.5	37.3 ± 1.1	36.2 ± 1.6	35.0 ± 0.6	35.2 ± 0.6	36.5 ± 2.0	38.5 ± 1.3
Globulin (g/L)	34.8 ± 1.0	35.3 ± 0.8	38.8 ± 2.2	30.5 ± 0.6	35.2 ± 0.4	34.7 ± 0.8	35.2 ± 1.0	33.0 ± 0.9
Bilirubin (*µ*mol/L)	1 ± 0	1 ± 0	1 ± 0	1 ± 0	1 ± 0	1 ± 0	1 ± 0	1 ± 0
ALP (iu/L)	307 ± 35	291.3 ± 19	301 ± 20	260 ± 23	270 ± 25	265 ± 22	367 ± 46	286 ± 16
AST (iu/L)	171 ± 33	149 ± 9	173 ± 27	135 ± 11	146 ± 9	115 ± 2	129 ± 11	116 ± 9
ALT (iu/L)	61 ± 6	57 ± 3	101 ± 19^∗*β*^	56 ± 3	69 ± 8	58 ± 3	63 ± 4	76 ± 4
GGT (iu/L)	0	0	0	0	0	0	0	0

Data were expressed as mean ± SEM. *P value < 0.05 is significant compared to control group;^ #^ *P* value < 0.05 is significant compared to baseline value; ^*β*^ *P* value < 0.05 is significant compared to other groups.
